# Protocol for the P3BEP trial (ANZUP 1302): an international randomised phase 3 trial of accelerated versus standard BEP chemotherapy for adult and paediatric male and female patients with intermediate and poor-risk metastatic germ cell tumours

**DOI:** 10.1186/s12885-018-4745-3

**Published:** 2018-08-29

**Authors:** Nicola J. Lawrence, Howard Chan, Guy Toner, Martin R. Stockler, Andrew Martin, Sonia Yip, Nicole Wong, Annie Yeung, Danish Mazhar, Farzana Pashankar, Lindsay Frazier, Ray McDermott, Roderick Walker, Hsiang Tan, Ian D. Davis, Peter Grimison, on behalf of ANZUP

**Affiliations:** 1 Australian and New Zealand Urogenital and Prostate Cancer Trials Group, Sydney, NSW Australia; 2 NHMRC Clinical Trials Centre, Lifehouse Level 6, 119–143 Missenden Road, Camperdown, NSW 2050 Australia; 3 Peter MacCallum Cancer Centre, University of Melbourne, Melbourne, Australia; 4 Chris O’Brien Lifehouse, 119–143 Missenden Road, Camperdown, NSW 2050 Australia; 5 Concord Cancer Centre, Concord Repatriation General Hospital, Sydney, NSW Australia; 6 Sydney Catalyst Translational Cancer Research Centre, University of Sydney, Camperdown, NSW Australia; 7 Cambridge University Hospitals NHS Foundation Trust, Cambridge, UK; 8 Yale University School of Medicine, New Haven, CT USA; 9 Dana-Farber Cancer Institute and Boston Children’s Hospital, Boston, MA USA; 10 Cancer Trials Ireland, Dublin, Ireland; 11 Lady Cilento Children’s Hospital, Brisbane, QLD Australia; 12 Princess Alexandra Hospital, Brisbane, QLD Australia; 13 Royal Adelaide Hospital, Adelaide, South Australia Australia; 14 Monash University Eastern Health Clinical School, Melbourne, VIC Australia

**Keywords:** Germ cell tumours, Phase 3 trial, Chemotherapy

## Abstract

**Background:**

Bleomycin, etoposide, and cisplatin (BEP) chemotherapy administered every 3 weeks for 4 cycles remains the standard first line treatment for patients with intermediate- and poor-risk metastatic germ cell tumours (GCTs). Administering standard chemotherapy 2-weekly rather than 3-weekly, so-called ‘accelerating chemotherapy’, has improved cure rates in other cancers. An Australian multicentre phase 2 trial demonstrated this regimen is feasible and tolerable with efficacy data that appears promising. The aim of this trial is to determine if accelerated BEP is superior to standard BEP as first line chemotherapy for adult and paediatric male and female participants with intermediate and poor risk metastatic GCTs.

**Methods:**

This is an open label, randomised, stratified, 2-arm, international multicentre, 2 stage, phase 3 clinical trial. Participants are randomised 1:1 to receive accelerated BEP or standard BEP chemotherapy. Eligible male or female participants, aged between 11 and 45 years with intermediate or poor-risk metastatic GCTs for first line chemotherapy will be enrolled from Australia, the United Kingdom and the United States. Participants will have regular follow up for at least 5 years. The primary endpoint for stage 1 of the trial (*n* = 150) is complete response rate and for the entire trial (*n* = 500) is progression free survival. Secondary endpoints include response following treatment completion (by a protocol-specific response criteria), adverse events, health-related quality of life, treatment preference, delivered dose-intensity of chemotherapy (relative to standard BEP), overall survival and associations between biomarkers (to be specified) and their correlations with clinical outcomes.

**Discussion:**

This is the first international randomised clinical trial for intermediate and poor-risk metastatic extra-cranial GCTs involving both adult and pediatric age groups open to both males and females. It is also the largest, current randomised trial for germ cell tumours in the world. Positive results for this affordable intervention could change the global standard of care for intermediate and poor risk germ cell tumours, improve cure rates, avoid the need for toxic and costly salvage treatment, and return young adults to long, healthy and productive lives.

**Trial registration:**

ACTRN 12613000496718 on 3rd May 2013 and Clinicaltrials.gov NCT02582697 on 21st October 2015.

## Background

The most common malignancy affecting adolescent and young adult males in Western countries is germ cell tumours (GCTs) [1]. Although most patients with good prognostic features have excellent outcomes, the cure rates for male patients with advanced disease and intermediate or poor prognostic features are only 79 and 48% respectively [2]. GCTs are rarer in females, however in females aged between 10 and 30 years they account for 70% of ovarian neoplasms [3].

The efficacy of first-line chemotherapy has not improved since the introduction of bleomycin, etoposide, cisplatin (BEP) in the mid-1980s. BEP chemotherapy given every 3 weeks for 4 cycles remain the global accepted standard of care for intermediate, and poor prognosis male patients [4]. Paediatric and female patients with GCTs are often not included in clinical trials due to the rarity of disease. The current management algorithms for these groups are based on extrapolations from other settings [3, 5].

Accelerating chemotherapy by administering the same doses more frequently has increased cure rates in other cancers, including breast cancer, lymphoma (prior to rituximab) and Ewing’s sarcoma [6–8]. The hypothesised mechanism is that accelerated chemotherapy with shorter cycles can overcome the rapid regrowth of shrinking tumours induced by chemotherapy [9, 10]. Accelerating chemotherapy is feasible with the development and availability of therapeutic granulocyte colony–stimulating factor (G-CSF) e.g. filgrastim, which reduces the duration of leukopenia [11]. Accelerated regimens may be preferable to patients as treatment is completed faster, it may improve compliance and has minimal additional financial cost.

A single arm phase 2 trial of 43 patients demonstrated that the regimen is feasible and tolerable [12]. The long term efficacy data appears promising with 5 year overall survival of 92% (95% CI 54% to 99%) for patients with poor prognostic features and 94% (95% CI 63% to 99%) for patients with intermediate prognostic features [13].

The aim of this phase 3 trial is to determine if accelerated BEP is superior to standard BEP as first-line chemotherapy for intermediate and poor-risk metastatic GCTs.

## Methods

### Study design

**Fig. 1:**
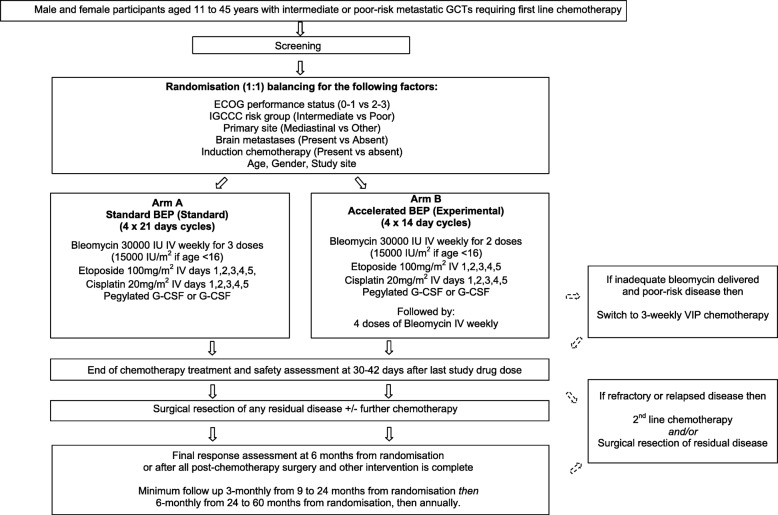
Study Schema

This trial is an open label randomised, 2-arm, multi-centre, phase 3 trial. Participants are randomised 1:1 to receive 4 cycles of either accelerated BEP chemotherapy given 2 weekly or standard BEP chemotherapy given 3 weekly (Fig. 1). Randomisation will be implemented using a minimisation approach balancing for; ECOG performance status (0–1 vs 2–3), International germ cell cancer consensus classification (IGCCC) risk group (intermediate vs poor), primary site (mediastinal vs other), brain metastases (present vs absent), induction chemotherapy (present vs absent), age (≥ 16 years vs < 16 years), gender (male vs female), and study site.

This international trial is led by the Australian and New Zealand Urogenital and Prostate Cancer Trials Group (ANZUP) in collaboration with the National Health and Medical Research Council Clinical Trials Centre (NHMRC CTC), Sydney, Australia. Key international collaborators include the Cambridge Clinical Trials Unit (United Kingdom), Children’s Oncology Group (United States) and Cancer Trials Ireland. Forty eight participants have been recruited from Australia and New Zealand since 2014, and 4 from the United Kingdom since opening to recruitment in 2017. The Children’s Oncology Group and Cancer Trials Ireland are planned to open to recruitment in the near future. The study will be performed in accordance with the Declaration of Helsinki and satisfy the regulatory requirements in Australia, United Kingdom and United States of America.

### Study objectives

The primary objective is progression-free survival (PFS), defined as from the date of randomisation until the criteria for disease progression are met or death. Secondary objectives include response following treatment completion (protocol specific criteria), adverse events (National Cancer Institute Common Terminology Criteria for Adverse Events version 4.03 [14]), health related quality of life (QLQ-C30 [15] and -TC-26 [16]), treatment preference, delivered dose-intensity of chemotherapy and overall survival. Tertiary objectives include exploratory studies of biomarkers and their correlations with clinical outcome. Initial response assessment is measured at the 30–42 day safety assessment. Final response assessment at 6 months from randomisation or after all post-chemotherapy surgery and other interventions are completed. Participants will continue regular follow-up for at least 5 years.

### Eligibility criteria

Key inclusion and exclusion criteria include age between 11 and 45 years, intermediate or poor prognosis germ cell tumour as defined by IGCCC (modified with different lactate dehydrogenase criteria for intermediate risk non-seminoma, and inclusion of ovarian primaries) and adequate organ function. Participants who need to start therapy urgently may commence study chemotherapy prior to registration and randomisation given the treatment is identical for the first 2 weeks and forms part of standard of care management. Such participants must be discussed with the coordinating centre prior to subsequent registration, and they must then be registered within 10 days of commencing chemotherapy. The full eligibility criteria are listed in Table 1.

**Table 1:** Eligibility Criteria

Inclusion Criteria	
1. Age ≥ 11 years and ≤ 45 years on the date of randomisation	
2. Histologically or cytologically confirmed germ cell tumour (non-seminoma or seminoma), or exceptionally raised tumour markers (AFP ≥ 1000 ng/mL and/or HCG ≥5000 IU/L) without histologic or cytologic confirmation in the rare case where pattern of metastases consistent with GCT, high tumour burden, and a need to start therapy urgently.	
3. Primary arising in testis, ovary, retro-peritoneum, or mediastinum	
4. Metastatic disease or non-testicular primary	
5. Intermediate or poor prognosis as defined by IGCC classification (modified with different LDH criteria for intermediate risk non-seminoma, and inclusion of ovarian primaries).	
6. Adequate bone marrow function with ANC ≥1.0 × 10^9^/L. Platelet count ≥100 × 10^6^/L	
7. Adequate liver function where bilirubin must be ≤1.5 x ULN, except participants with Gilbert’s syndrome or if the elevations are due to hepatic metastases.	
8. Adequate renal function	
9. ECOG performance status of 0–3	
10. Study treatment both planned and able to start within 14 days of randomisation	
11. Willing and able to comply with all study requirements, including treatment, timing and nature of required assessments.	
12. Able to provide signed, written informed consent	
Exclusion criteria	
1. Other primary malignancy	
2. Previous chemotherapy or radiotherapy, except	
a. Pure seminoma that relapsed after adjuvant radiotherapy or chemotherapy with 1–2 cycles of cisplatin	
b. Non-seminoma and poor prognosis by IGCCC criteria in the rare case where low dose induction chemotherapy is given prior to registration because patients is not fit enough to receive protocol chemotherapy.	
c. Participants who need to start therapy urgently prior to completing study-specific baseline investigations	
3. Significant cardiac disease	
4. Significant co-morbid respiratory disease	
5. Peripheral neuropathy ≥ grade 2 or clinically significant sensorineural hearing loss	
6. Concurrent illness that prevent the completion of the interventions listed in the protocol	
7. Participants who are sexually active and are not willing to use an effective contraceptive method during this study.	
8. Known allergy or hypersensitivity to any of the study drugs	
9. Presence of any psychological, familial, sociological or geographical condition that the investigators believe will lead to compliance issues.	

### Treatment

The experimental arm is accelerated BEP given as bleomycin 30,000 international units (IU) (15,000 IU/m^2^ in participants aged less than 16) intravenous (IV) weekly on day 1 and 8, etoposide 100 mg/m^2^ on days 1–5 and cisplatin 20 mg/m^2^ on days 1–5 every 2 weeks for 4 cycles, followed by single agent bleomycin 30,000 IU (15,000 IU/m^2^ in participants aged less than 16 years) IV once a week for a further 4 weeks to a total of 12 doses of bleomycin. The control arm is standard BEP given as bleomycin 30,000 IU (15,000 IU/m^2^ in participants aged less than 16) IV weekly on day 1, 8 and 15, etoposide 100 mg/m^2^ on days 1–5 and cisplatin 20 mg/m^2^ on days 1–5 every 3 weeks for 4 cycles. G-CSF support is given in both treatment arms.

Every attempt should be made to deliver chemotherapy at full dose and without delay from the planned schedule, as dose and dose-intensity are important predictors of outcome. Dose reductions for etoposide are specified in the protocol. There are no dose reductions for cisplatin or bleomycin allowed. Study treatment will be permanently discontinued for unacceptable toxicity, delay of day 1 of treatment for more than 21 days due to treatment-related adverse events, unequivocal progression, occurrence of an exclusion criteria or illness affecting participant safety, failure to comply with the protocol or if the investigator does not think it is in the participant’s best interest to continue. If a participant develops pulmonary toxicity then bleomycin should be stopped. If the participant has poor risk disease and less than 8 doses of bleomycin have been administered then the participant should stop BEP, and ifosfamide and mesna should be used with cisplatin and etoposide, as per the etoposide, ifosfamide, cisplatin (VIP) regimen. Surgical resection of residual masses and subsequent treatment following the completion of chemotherapy are specified in the protocol.

### Assessment schedule

Participants are assessed at baseline, prior to each cycle of chemotherapy, at completion of study treatment, then at 6, 9, 12, 18, 24, 30, 36, 42, 48, 54 and 60 months from randomisation (Table 2). Assessments at each time point include performance status, adverse events, blood tests (blood count, biochemistry, tumour markers), quality of life (up to 12 months), lung function tests (for Australian sites up to 12 months), CT imaging (at baseline; after randomisation at 4, 12, 24 and 60 months; and as clinically indicated), disease status, subsequent treatment and survival. Biospecimens including tumour tissue (formalin-fixed paraffin-embedded) and blood (whole blood and plasma) at baseline will be collected from consenting participants for use in future translational research.

**Table 2:** Schedule of Assessments

Visit	Baseline	On treatment: BEP chemotherapy Cycles 1 to 4		End of BEP chemotherapy safety assessment (Initial response assessment)	Final response assessment	Follow-up until progression	Follow-up after progression
	Within 21 days prior to randomisation	Day 1 of Cycle (or within 3 days)	Day 8 and 15 of Cycle (or within 3 days)	30–42 days after the last dose of study treatment	6 months from randomisation, or after completion of all post-chemo surgery and other interventions (± 1 month)	9, 12, 15, 18, 21, 24, 30, 36, 42, 48, 54 and 60 months from randomisation, then annually (± 1 month)	Every 6 months (± 1 month)
Clinical assessment	X	X		X	X	X (until 60 months)	
Respiratory symptoms/signs	X	X	X	X			
Adverse Event		X		X			
Blood tests including tumour markers	X	X	X	X	X	X (until 60 months)	
CT imaging	X			X	X	X (12, 24, 36, 60 months)	
Chest X-Ray	X	X		X			
Patient-Rated Measures		X		X	X	X (9, 12, 18 months)	
Translational blood and tissue	Optional						
Patient Status	X			X	X	X	X

### Statistical analysis

Stage 1 of the study will recruit 150 participants (75 per arm) which will provide 80% power at the 5% level of significance to detect an improvement in the favourable response rate from 59% with standard BEP to 80% with accelerated BEP. If results from Stage I are promising, Stage 2 of the study will recruit an additional 350 participants for a total sample size of 500 participants. A study of 500 patients followed until 140 PFS events are observed will provide > 80% power at the 5% level of significance to detect a hazard ratio of 0.6. An effect of this size corresponds to a 7% improvement in PFS at 2 years from 81% with standard BEP to 88% with accelerated BEP.

## Discussion

The results of this study will determine if accelerated BEP chemotherapy is superior to standard BEP chemotherapy in the first-line treatment of intermediate and poor-risk metastatic GCTs. The collection of biospecimens will allow for future translational research studies to determine associations between biomarkers (to be specified) and their correlations with clinical outcomes. This is the first international randomised clinical trial for intermediate and poor-risk metastatic extra-cranial GCTs involving both adult and pediatric age groups open to both males and females.

## Abbreviations

ANZUP: Australian and New Zealand Urogenital and Prostate Cancer Trials Group
BEP: Bleomycin, etoposide, and cisplatin
G-CSF: Granulocyte colony–stimulating factor
GCT: Germ cell tumour
IGCCC: International Germ Cell Consensus Classification
IU: International units
IV: Intravenous
NHMRC CTC: National Health and Medical Research Council Clinical Trials Centre
PFS: Progression-free survival

## References

[1] Howlader NNA, Krapcho M, Miller D, Bishop K, Kosary CL, Yu M, Ruhl J, Tatalovich Z, Mariotto A, Lewis DR, Chen HS, Feuer EJ, Cronin KA (2017). SEER Cancer Statistics Review, 1975-2014.

[2] International Germ Cell Cancer Collaborative Group (1997). International Germ Cell Consensus Classification: a prognostic factor- based staging system for metastatic germ cell cancers. International germ cell Cancer collaborative group. J Clin Oncol.

[3] Meisel JL, Woo KM, Sudarsan N, Eng J, Patil S, Jacobsen EP (2015). Development of a risk stratification system to guide treatment for female germ cell tumors. Gynecol Oncol.

[4] Grimison PS, Toner GC, Scardino PT, Linehan WM, Zelefsky MJ, Vogelzang NJ, Rini BI, Bochner BH (2011). Management of Advanced Germ Cell Tumors: IGCCC Intermediate- and Poor-Risk Patients. Comprehensive Textbook of Genitourinary Oncology.

[5] Murugaesu N, Schmid P, Dancey G, Agarwal R, Holden L, McNeish I (2006). Malignant ovarian germ cell tumors: identification of novel prognostic markers and long-term outcome after multimodality treatment. J Clin Oncol.

[6] Bonilla L, Ben-Aharon I, Vidal L, Gafter-Gvili A, Leibovici L, Stemmer SM (2010). Dose-dense chemotherapy in nonmetastatic breast cancer: a systematic review and meta-analysis of randomized controlled trials. J Natl Cancer Inst.

[7] Pfreundschuh M, Trumper L, Kloess M, Schmits R, Feller AC, Rudolph C (2004). Two-weekly or 3-weekly CHOP chemotherapy with or without etoposide for the treatment of young patients with good-prognosis (normal LDH) aggressive lymphomas: results of the NHL-B1 trial of the DSHNHL. Blood.

[8] Womer RB, West DC, Krailo MD, Dickman PS, Pawel BR, Grier HE (2012). Randomized controlled trial of interval-compressed chemotherapy for the treatment of localized Ewing sarcoma: a report from the Children's oncology group. J Clin Oncol.

[9] Gregory SA, Trumper L (2005). Chemotherapy dose intensity in non-Hodgkin's lymphoma: is dose intensity an emerging paradigm for better outcomes?. Ann Oncol.

[10] Simon R, Norton L (2006). The Norton-Simon hypothesis: designing more effective and less toxic chemotherapeutic regimens. Nat Clin Pract Oncol.

[11] Kim JJ, Tannock IF (2005). Repopulation of cancer cells during therapy: an important cause of treatment failure. Nat Rev Cancer.

[12] Grimison PS, Stockler MR, Chatfield M, Thomson DB, Gebski V, Friedlander M (2014). Accelerated BEP for metastatic germ cell tumours: a multicenter phase II trial by the Australian and New Zealand urogenital and prostate Cancer trials group (ANZUP). Ann Oncol.

[13] Lawrence N, Martin A, Toner G, Stockler M, Buizen L, Thomson D (2016). Long-term outcomes of accelerated BEP (bleomycin, etoposide, cisplatin) for advanced germ cell tumours: updated analysis of an Australian multicentre phase II trial by the Australian and New Zealand urogenital and prostate Cancer trials group (ANZUP). Ann Oncol.

[14] National Cancer Institute Cancer Therapy Evaluation Program (CTEP) (2009). Common Toxicity Criteria for Adverse Events v4.03 (CTCAE).

[15] Aaronson NK, Ahmedzai S, Bergman B, Bullinger M, Cull A, Duez NJ (1993). The European-organization-for-research-and-treatment-of-Cancer Qlq-C30 - a quality-of-life instrument for use in international clinical-trials in oncology. J Natl Cancer Inst.

[16] Holzner B, Efficace F, Basso U, Johnson CD, Aaronson NK, Arraras JI (2013). Cross-cultural development of an EORTC questionnaire to assess health-related quality of life in patients with testicular cancer: the EORTC QLQ-TC26. Qual Life Res Int J Qual Life Asp Treat Care Rehab.

